# Inequivalent Solvation Effects on the N 1s Levels of Self-Associated Melamine Molecules in Aqueous Solution

**DOI:** 10.1021/acs.jpcb.3c00327

**Published:** 2023-03-27

**Authors:** Aurora Ponzi, Marta Rosa, Gregor Kladnik, Isaak Unger, Alessandra Ciavardini, Lorys Di Nardi, Elisa Viola, Christophe Nicolas, Nađa Došlić, Andrea Goldoni, Valeria Lanzilotto

**Affiliations:** † Division of Physical Chemistry, Ruđer Bošković Institute, 10000 Zagreb, Croatia; ‡ Department of Chemical Sciences, University of Padova, 35122 Padova, Italy; § Department of Physics, University of Ljubljana, 1000 Ljubljana, Slovenia; ∥ IOM-CNR, Laboratorio TASC, Basovizza SS-14, Km 163.5, 34149 Trieste, Italy; ⊥ Department of Physics and Astronomy, Uppsala University, 751 20 Uppsala, Sweden; # CERIC-ERIC, 34149 Trieste, Italy; ∇ Department of Chemistry, Sapienza University of Rome, 00185 Roma, Italy; ○ Synchrotron SOLEIL, 91192 Paris, France; ◆ Elettra Synchrotron, Micro & Nano Carbon Laboratory, 34149 Trieste, Italy

## Abstract

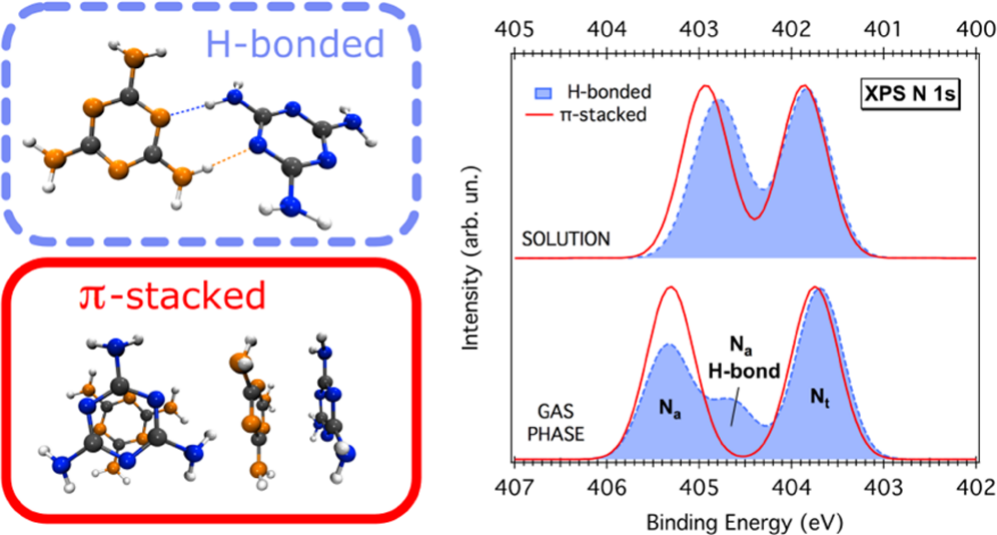

This work shows how the N 1s photoemission (PE) spectrum of self-associated melamine molecules in aqueous solution has been successfully rationalized using an integrated computational approach encompassing classical metadynamics simulations and quantum calculations based on density functional theory (DFT). The first approach allowed us to describe interacting melamine molecules in explicit waters and to identify dimeric configurations based on π–π and/or H-bonding interactions. Then, N 1s binding energies (BEs) and PE spectra were computed at the DFT level for all structures both in the gas phase and in an implicit solvent. While pure π-stacked dimers show gas-phase PE spectra almost identical to that of the monomer, those of the H-bonded dimers are sensibly affected by NH···NH or NH···NC interactions. Interestingly, the solvation suppresses all of the non-equivalences due to the H-bonds yielding similar PE spectra for all dimers, matching very well our measurements.

## Introduction

Melamine (triamino-*s*-triazine) and its condensed derivative melem (triamino-*s*-heptazine) can be regarded as the building blocks of a series of CNH-containing polymers (p-CNH), also known as carbon nitrides, which have proven to produce H_2_ from water under visible light irradiation.^1,2^ Representative CNH-polymers are polytriazine-imide, featuring a graphitic layered structure,^3,4^ and melon, a linear polymer that can organize in tightly H-bonded two-dimensional (2D) domains further stabilized by interlayer van der Waals interactions (see Supporting Information Figure S1).^5^

p-CNH are usually considered as wide-bandgap semiconductors (2.5–2.8 eV),^2^ and an understanding of their photocatalytic activity brings into play the photogeneration of excitons, followed by their dissociation in free charges, which migrate toward the polymer–liquid interface to drive the redox reactions (i.e., reduction of protons and oxidation of water).^6,7^ According to the semiconductor model, a relationship between photoactivity and the polymer structure should be expected, as an ordered or crystalline phase should promote the charge separation and transport required for the aforementioned reactions.^8^ However, this is not observed, and it is often justified by the fact that crystalline phases do not sufficiently expose the active sites for H-binding the water molecules.^9,10^ In this regard, Lotsch et al. identified primary (−NH_2_) and secondary amino (−NH−) groups as the main active sites.^10^ Specifically, amino groups would allow better coordination of the platinum cocatalyst, which is usually loaded to the polymer to facilitate interfacial charge transfer.

Contrary to the semiconductor model, recent theoretical investigations have shown that the water-splitting reaction with p-CNH may be described as a photochemical reaction essentially confined on a single triazine or heptazine unit.^11−14^ In this case, the active site of the reaction is attributed to the pyridine-like nitrogen (−N=C) by which a photo-induced proton-coupled electron transfer (PCET) occurs from the H-bonded water molecule to the heterocycle, yielding two neutral radicals: the OH radical and a hypervalent heterocycle radical (N-hydrogenated heterocycle). Successively, the excess H can be photo-detached from the heterocyclic radical, or two heterocyclic radicals can recombine via an exothermic dark reaction to produce molecular hydrogen. Although the formation of hydroxyl radicals has been indirectly observed through chemical scavenging and spectroscopic detection (laser-induced fluorescence) of hydroxylated products,^15^ the semiconductor model is most widely accepted, and it is driving the synthetic strategies aimed at ameliorating the photocatalytic properties of p-CNH and other polymeric materials.^7^

A more direct and unambiguous way to identify reaction intermediates, such as OH and/or hypervalent heterocyclic radicals, is to exploit the chemical sensitivity of techniques like X-ray photoemission spectroscopy (XPS), which is able to finely assess the oxidation state of light-weight elements (C, N, O). For instance, we successfully used XPS for studying the water–melamine interaction by dosing water, in ultra-high vacuum conditions (UHV), on a monolayer of melamine molecules adsorbed on Cu(111).^16^ Upon water uptake, a 1:2 water–melamine complex is formed, where one melamine acts as H-donor (NH···OHH), and the other one acts as H-acceptor (C=N···HOH). This H-bonded configuration significantly lowers the N 1s binding energy (BE) of the amino-N (−NH_2_) while leaving almost unperturbed the BE of the triazine-N (C=N). We envisage that more dramatic core-level shifts will be observed both in the N 1s (for the organic catalyst) and the O 1s (for the water molecule) photoemission spectra if the PCET reaction is going to take place.

With the perspective to perform *operando* core-level spectroscopy experiments, we employed the XPS μ-liquid jet (XPS μ-LJ) technique for directly probing the local electronic state of the melamine N-functional groups in aqueous solution, the natural environment of the PCET reaction. Additionally, ultraviolet (UV) absorption spectroscopy measurements were performed on melamine solutions, revealing important self-association phenomena. Hence, a proper understanding of the XPS μ-LJ data required an in-depth investigation on how the molecule’s BEs are affected by the water–melamine and melamine–melamine interactions. In the framework of core-level spectroscopies, an attempt to disentangle the solute–solute and solute–water contributions has been only reported for aqueous imidazole, whose N 1s near-edge X-ray absorption fine structure (NEXAFS) spectra were interpreted by considering several arrangements of microhydrated monomeric and trimeric structures.^17 ,18^ This study has, therefore, a double goal. The first goal is to rationalize the N 1s photoemission spectrum of aqueous melamine, in particular, the experimentally observed splitting between the amino-N (N_a_) and the triazine-N (N_t_) energy levels. Our second goal is to propose a generally applicable and efficient computational protocol for evaluating the impact of different types of interactions (molecule–molecule and molecule–water) on the core-level spectra of organic molecules in aqueous solution. The protocol combines classical metadynamics sampling with quantum calculations based on density functional theory (DFT). On the one hand, metadynamics simulations allowed us to describe melamine molecules interacting in explicit waters and to explore different interaction free-energy minima on time scales that are not accessible by classical molecular dynamics. On the other hand, quantum calculations were performed in implicit solvent for each of the relevant configurations. This allowed us to keep the polarization effect of the solvent in the description and to obtain accurate structural and spectral results. Specifically, we found that solvation affects π-stacked and H-bonded structures in rather different ways (vide infra), which paradoxically makes their spectra quite similar.

## Experimental Methods

### PE Measurements

Photoemission spectroscopy studies of melamine aqueous solutions were performed at the PLEIADES beamline of the synchrotron radiation facility SOLEIL (Paris, France). Details of the experimental setup have been reported previously.^19^ Briefly, the liquid samples are injected into an evacuated experimental chamber through a 40 μm diameter glass capillary at a typical flow rate of 0.8 mL min^–1^. The propagation of the synchrotron light beam is perpendicular to both the liquid jet and the electron detection axis of the spectrometer. Light polarization was set parallel to the spectrometer axis.

Samples were prepared freshly from commercially obtained melamine (Sigma-Aldrich, purity 99%) and demineralized water (18.2 MΩ, Millipore Direct-Q). Measurements were performed for 26 mM (millimolar) solutions, which approximately correspond to the highest obtainable concentration at 293 K (3.24 g melamine/1 L H_2_O).^20^ All samples were sonicated to facilitate solubilization and filtered to remove solid particles, which may disturb the flow in the liquid jet or cause the injection system to fail. For all measurements, NaCl salt (50 mM) was added to the samples to maintain electrical conductivity and mitigate potentially deleterious sample charging effects.^21,22^ This is common practice when measuring PE spectra from liquid water.^23^

The N 1s PE spectrum was collected using a photon energy of 500 eV, as it ensures both high photoionization probability and a constant/linear background around the photoemission peak. The same photon energy was used to collect the valence PE spectrum, which was needed to calibrate the binding energy scale by aligning the 1b_1_ PE line of liquid water to 11.16 eV.^24^ At this photon energy, the kinetic energy range of the N 1s electrons (95–99 eV) nearly corresponds to the minimum electron attenuation length (EAL), which makes the measurements highly surface-sensitive (i.e., 63% of primary photoelectrons originate from a layer of thickness equal to EAL). The actual estimate of the minimum EAL ranges from 5–10 Å^25,26^ up to 20 Å.^27^

Similarly to previous studies on glycine,^28^ the core PE spectra of solvated melamine were compared to those acquired for gaseous melamine, the latter being already published by some of us.^29^ Gas-phase (GP) spectra were deconvoluted using bi-Gaussian functions, which are usually used for fitting asymmetric PE peaks of gaseous species.^30−33^ The ratio between the N_a_ and N_t_ areas (N_a_/N_t_) is found to be approx. 1.1, slightly in favor of the amino-N component. On the other hand, liquid jet (LJ) PE spectra were fitted using Voigt profiles having common Gaussian and Lorentzian broadening. The latter was constrained between 0.10 and 0.13 eV.^34^ The area ratio (N_a1_ + N_a2_)/N_t_ was found to be approx. 1.3, slightly larger with respect to the gas-phase value. This could be attributed to shadowing effects derived from some preferential molecular ordering occurring at the liquid/vacuum interface.

### UV Absorption Measurements

The hypochromic effect in UV absorption spectra of melamine in aqueous solution was investigated over a wide concentration range (0.020 μM to 1.0 mM), according to the following procedure. A stock solution (25 mM) of melamine in Millipore grade water was prepared by weight, and dilutions of this stock were used for absorption measurements. UV spectra were recorded on a Varian Cary 50 spectrophotometer in the wavelength range of 200–350 nm using quartz cuvettes with a path length of 1.00, 0.200, or 0.100 cm, depending on the solution concentration. Three independent dilution experiments were carried out to verify the reproducibility of the observed hypochromic effect.

## Theoretical Calculations

With the aim of studying the behavior of melamine molecules in solution, we devised a multiscale computational approach that exploits both classical and quantum methodologies. First, we used metadynamics to perform classical enhanced sampling simulations in explicit solvent, which allowed us to identify the most relevant interacting configurations of melamine in solution (see the Metadynamics Calculations Subsection). The presence of the explicit solvent allowed us to account not only for its polarization effect but also for its steric hindrance and for its ability to create hydrogen bonds with melamine molecules, together with its entropic effect, which cannot be neglected in free-energy calculations. Then, DFT calculations were performed for each of the relevant interacting configurations to obtain BEs and PE spectra (see the DFT Calculations Subsection).

### Metadynamics Calculations

The calculations were performed with the Gromacs 4.5.5 MD simulation package,^35,36^ complemented by the Plumed 2.0^37^ plug-in for metadynamics. Trajectory analyses were performed with Gromacs tools and VMD. The simulations were performed in explicit solvent using the TIP3P water model while the melamine force field (FF) was generated with the antechamber tool, and RESP charges were calculated with the RESP ESP charge Derive (R.E.D.) program. The quantum optimization calculation used as the basis for the R.E.D. calculation was performed with Gaussian 09, with a PBE/6-311g(d,p) basis set.

The volume of the simulation box was 5 × 5 × 15 nm^3^, while the water slab was 5 × 5 × 5 nm^3^. Periodic boundary conditions (PBC) were used. Two metadynamics simulations were performed: one with two melamine molecules and the other with four melamine molecules. In both simulations, the melamine molecules’ centers of mass (CMs) were not allowed to be farther than 2.5 nm. In this way, we kept the distance between neighboring replicas larger than 2 nm, which ensures negligible spurious interactions.

The integration time step was 1 fs, and all bonds were treated as holonomic constraints using the LINCS algorithm. The simulations were carried out in the NVT ensemble using a velocity rescaling thermostat^38^ at *T* = 300 K. The particle mesh Ewald (PME)^39^ electrostatic summation was used with a real-space cutoff of 1.2 nm.

Well-tempered metadynamics^40−42^ was used to sample the interaction configurations of melamine molecules by defining one or more collective variables (CVs). In metadynamics, the free-energy surface (FES) along a few CVs is sampled with the aid of a biasing Gaussian potential that fills the FES local minima, thus allowing the system to overcome free-energy barriers.

The widths of the Gaussian functions were initially set to 0.1 nm; the height was set to 2 kJ/mol. The bias potential was regularly updated every 2 ps. The well-tempered bias factor was 30 in all simulations.^42^ The two-molecule simulation was carried out by defining one CV as the distance between the CMs of the two melamine molecules. It was run until convergence of the FES was achieved after 50 ns. The four-molecule simulation was performed by pairing them in two couples and defining three CVs: CM1 and CM2 controlling the distance between melamine molecules belonging to the same couple and CM-pairs accounting for the distance between centers of mass of the two pairs. It was carried on for 180 ns, long enough to properly identify the most relevant interaction configurations and compare them with the dimers configurations, even if convergence was not achieved.

### DFT Calculations

Starting from the relevant configurations obtained from metadynamics simulations, the PE spectra were calculated with the delta self-consistent field (ΔSCF) method, based on Kohn–Sham density functional theory (KS-DFT).^43^ In this approach, the core-level binding energies are calculated as the total energy difference between the neutral and the ionized system. To simulate the PE spectra in aqueous solution, the water solvent was included by implicit solvation using the COSMO model.^44^ Initial and final state electronic energies of melamine monomers and dimers, both in gas and aqueous phases, were calculated at the ground-state geometries obtained from MP2/cc-pVDZ calculations. For the case of aqueous phase, ground-state geometry optimization was performed within the COSMO field.

The BEs were determined with the B3LYP exchange–correlation functional. We used def2 valence triple-ζ plus polarization (def2-TZVPP) basis sets for all atoms except for the one (N) with the core hole, for which we used def2-QZVPP instead.

All calculations were performed with the Molpro package,^45^ apart from the geometry optimization that was performed with Turbomole.^46^

## Results and Discussion

### N 1s Photoemission Measurements

**Figure 1:**
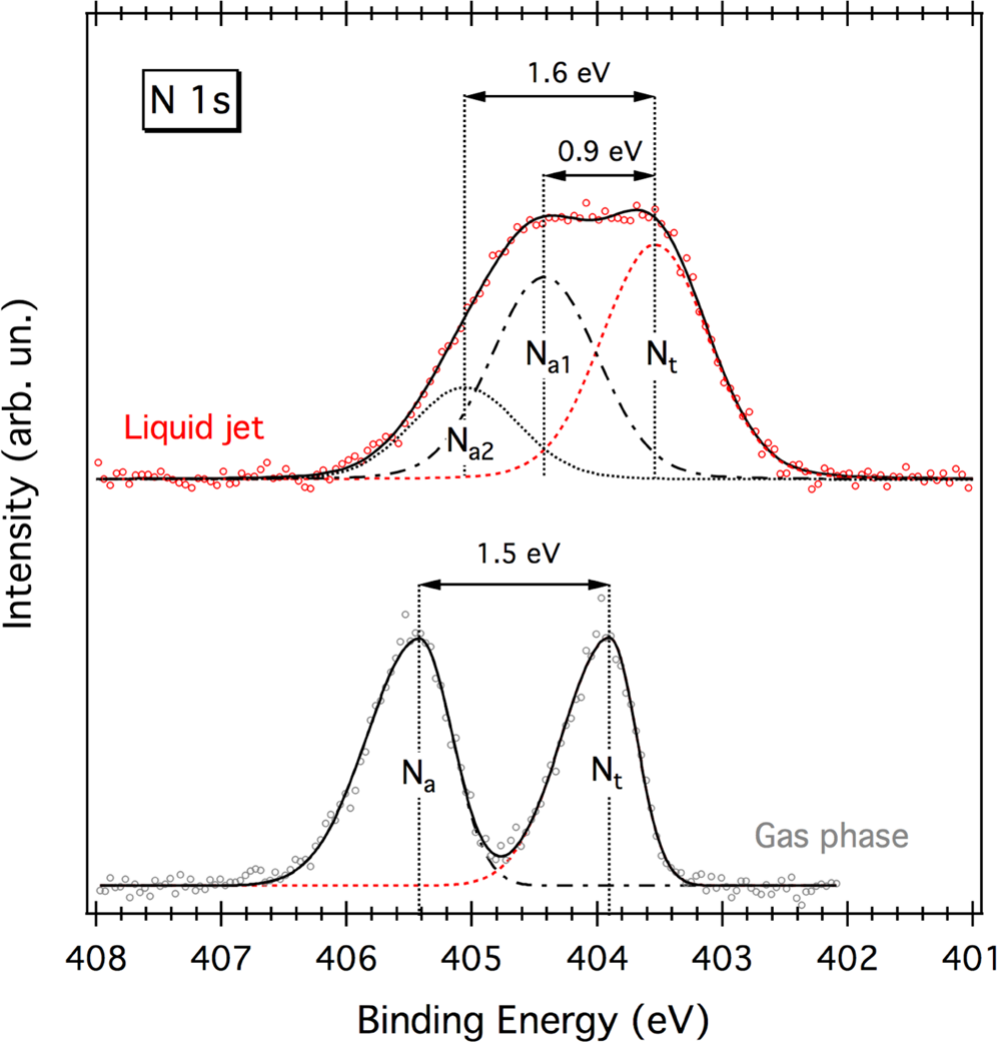
N 1s PE spectra of gaseous (“Gas phase” gray curve) and 26 mM melamine aqueous solution (“Liquid jet” red curve). Circles represent experimental data points; solid and dashed lines represent total fits and individual fits components, respectively. The gas-phase spectrum is adapted from ref (29), by permission from John Wiley & Sons Ltd.

N 1s PE spectra of melamine in gas phase and in aqueous solution (26 mM) are shown in Figure 1, while BEs and chemical shifts (*cs*) are summarized in Table 1. Peak fits were performed according to the lines described in the Experimental Methods Section. Gas-phase spectra are taken from our previous publication,^29^ where experimental details can be found. As shown, the gas-phase spectrum is characterized by two well-defined peaks of almost equal intensity separated by 1.5 eV. The peak at lower BE, 403.9 eV, arises from the triazine-N (N_t_), while the one at 405.4 eV is assigned to the amino-N (N_a_). On the other hand, the liquid jet curve features a single, rather broad peak, where at least three components can be identified: two main peaks at 403.5 eV (N_t_) and 404.4 eV (N_a1_) and a smaller one at 405.1 eV (N_a2_). Compared to the gas phase, the chemical shift between the two main components, N_t_ and N_a1_, in solution, is significantly smaller (0.9 eV), while the one between N_t_ and N_a2_ (1.6 eV) is almost unchanged. In solid-state samples, a *cs* of 0.9 eV is commonly observed for melamine H-bonded networks grown on Au(111), where a double H-bond of the type NH···N=C is responsible for a lowering of the amino-N BE leaving almost unperturbed that of the triazine-N.^28^ Conversely, a gas-phase-like *cs* can be observed for standing-up melamine molecules on Cu(111), where at least one amino group is pointing toward the vacuum side and therefore not involved in any specific molecule–molecule or molecule–substrate interaction.^16,47^ Such findings suggest that the μ-LJ spectrum is likely contributed by two different types of amino-N: those which are more affected by the local environment (N_a1_), and those which are less perturbed (N_a2_).

**Table 1:** Summary of the Experimental N 1s BEs and Chemical Shifts (*cs*) for Melamine in the Gas Phase and in Aqueous Solution/Liquid Jet (26 mM)

**Gas phase**			**Liquid jet**		
**N 1s level**	**BE (eV)**	***cs* (eV)**	**N 1s level**	**BE (eV)**	***cs* (eV)**
N_t_	403.9	-	N_t_	403.5	-
N_a_	405.4	1.5	N_a1_	404.4	0.9
			N_a2_	405.1	1.6

### Ultraviolet Absorption Measurements

**Figure 2:**
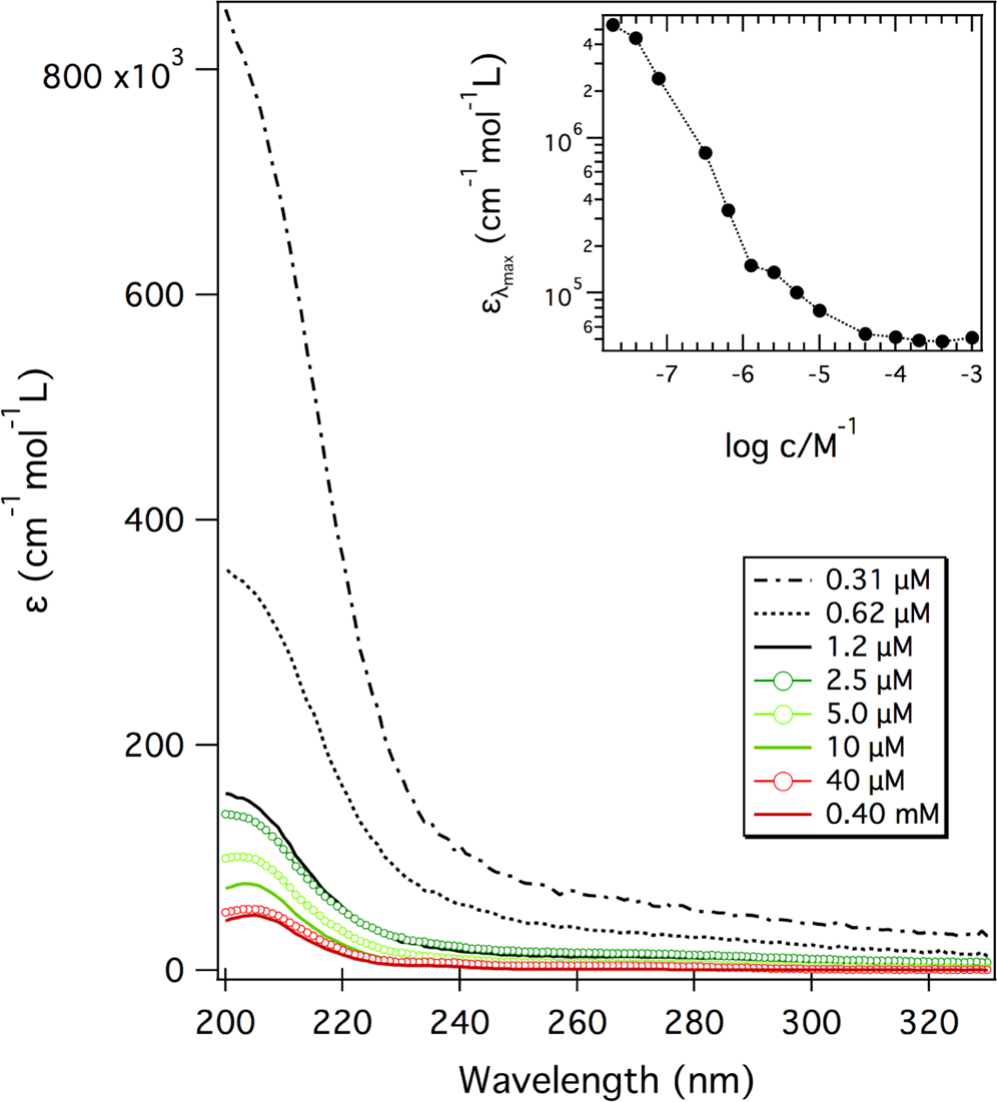
UV spectra (normalized for the concentration and optical path) acquired for melamine aqueous solutions in the 0.31 μM to 0.40 mM concentration range. The inset shows the molar absorption coefficient (ε) for the absorption maximum (ca. 204 nm) for a wider concentration range.

To get an in-depth understanding of the μ-LJ spectrum, we investigated first whether, at the concentrations used in our experiments, melamine exists as solvated monomers or rather as dimers or higher-order oligomers. For this purpose, we studied the changes in the UV absorption spectrum of melamine solutions by varying concentrations between 0.020 μM and 1.0 mM. Selected UV spectra (0.31 μM to 0.40 mM) are shown in Figure 2. The inset shows the apparent molar absorption coefficient (ε) at λ_max_ (ca. 204 nm) for the full range of explored concentrations.

As observed, the absorption maximum strongly decreases by 2 orders of magnitude (5 × 10^6^ to 5 × 10^4^ cm^–1^ mol^–1^ L) with increasing concentration until reaching a steady value at 40 μM. This large ε variation is accompanied by only a slight red shift of the band. Such a deviation from the Lambert–Beer law, known as the hypochromic effect, was previously observed for analogue N-heterocycles (pyridine, pyrimidine, imidazole, and their derivatives) and interpreted in terms of self-association phenomena.^48−50^ Self-association can occur either by means of π–π interactions or via intermolecular H-bonds, the latter being favored by ad-hoc functionalizations or adjustment of the solution pH.

Actually, in a previous study, Chattaraj et al.^51^ performed isothermal titration calorimetry experiments for a 20 mM melamine solution, showing an enthalpy-driven 1:1 binding of melamine monomers. We analyzed the variation of the melamine ε as a function of concentration and estimated the self-association constant (*K*) for melamine dimerization using the approach of Morcillo et al.^52^ The obtained value (*K* = 10^20^) is very high if placed in the context of N-heterocycle self-association phenomena in aqueous solutions. Taking into account the limitations of the method and of the model, this calculation provides an order of magnitude estimation of the constant and suggests that melamine shows a strong tendency to self-associate, even in very dilute solutions.

### Metadynamics Simulations

Given the strong hypochromic effect observed in the UV spectra, it was deemed that a better understanding of the interactions governing the arrangements of melamine molecules in water was needed to interpret the μ-LJ chemical shifts. With this aim, we performed a multiscale computational study involving both classical and quantum simulations. By exploiting classical molecular dynamics (MD) simulations in explicit waters, we identified the possible interaction configurations of melamine molecules. Then, the classical geometries were relaxed with the MP2 method and used to compute the core-level BEs (and PE spectra) with DFT using the COSMO continuum solvation model (see the Theoretical Calculations Section).

The exploration of free-energy surfaces (FES) of molecular aggregates in solution using classical MD is computationally very expensive as the presence of high free-energy barriers can trap the system in local minima. For this reason, we performed metadynamics simulations (see the Theoretical Calculations Section) that allow us to study the phenomena occurring on time scales that are not reached within unbiased classical MD simulations. More precisely, two sets of metadynamics simulations were carried out. In the first, we included two, and in the second, four melamine molecules. The latter allowed us to study the formation of higher-order structures such as trimers and tetramers, but it was found that these structures correspond mostly to combinations of dimeric structures (vide infra). Since the two-melamine system accounts for almost all fundamental interaction modes, BEs were calculated only for the dimeric structures.

**Figure 3:**
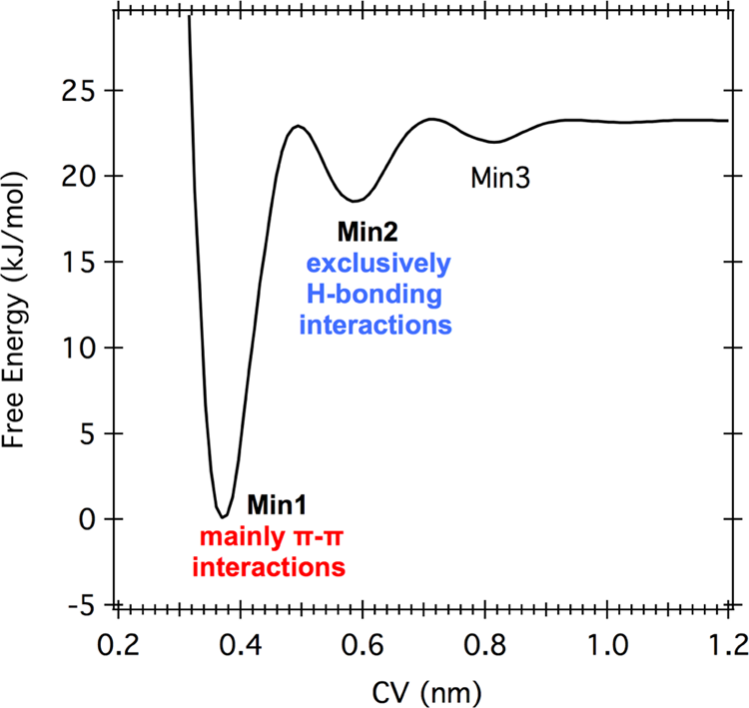
Free-energy surface of the two melamine metadynamics simulations. On the *x*-axis is the collective variable (CV), the distance between the center of mass of the two molecules.

Figure 3 shows the FES of the two melamine molecules. The metadynamics simulation was performed with a constraint that kept the two moieties within 2.5 nm from the water/vacuum interface to take into account the surface sensitivity of the XPS μ-LJ experiment (see the Experimental Methods Section). As shown, the system has one strongly preferred interacting configuration, denoted Min1, and two other interacting minima (Min2, Min3), found higher in energy. The isolated, fully solvated configuration is the least probable. The most likely configuration is the one where π–π interactions are prevalent (Min1), while the second minimum (Min2) corresponds to dimers exclusively based on H-bonding interactions. Finally, the third minimum (Min3) corresponds to an interaction configuration mediated by a water molecule.

The free-energy values show that only Min1 is statistically relevant, covering more than 0.9 of the population. Nevertheless, Min2 geometries could be more relevant when more than two melamine molecules are present, as one can infer from the four-molecule metadynamics. Moreover, we know that quantum corrections can modify the relative depth of classical free-energy minima.^53^ For these reasons, we decided to extract representative geometries from both Min1 and Min2 and relaxed them with the MP2 method to find accurate quantum local minima within each basin.

**Figure 4:**
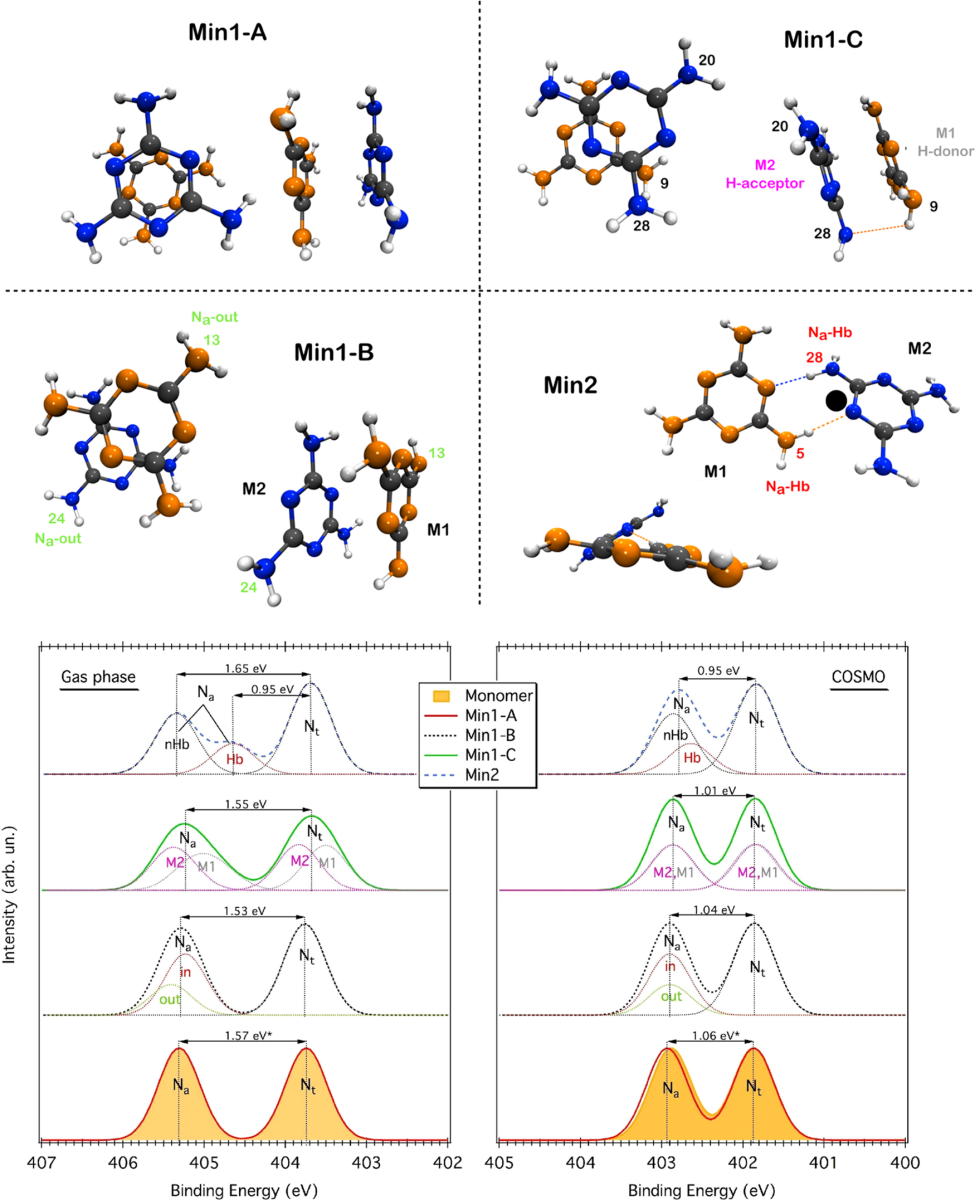
(Top panel): the gas-phase MP2/cc-pVDZ-optimized structures of the melamine dimers corresponding to Min1 and Min2 of the FES curve. For all structures, top and side views are reported. (Bottom panel): N 1s PE spectra simulated for the dimers reported in the upper panel, both in gas phase and in solution (COSMO). The N 1s spectra of the monomer (filled yellow curves) are also reported and shifted by −0.08 eV (gas phase) and +0.07 (COSMO) to match the N_t_ component of Min1-A. (*) For the sake of clarity, only the *cs* of the dimer is reported.

Three slightly different π-stacked configurations, labeled as Min1-A, Min1-B, and Min1-C, have been extracted from the Min1 structure, while for Min2, only one configuration is obtained. Their gas-phase MP2/cc-pVDZ-optimized geometries are shown in the top panel of Figure 4. A detailed description of the different geometries is reported in Supporting Information. Here, we present only the main structural features. In Min1-A, one ring is on top of the other, while in Min1-B and Min1-C, there is a slight displacement between the two rings. In addition, in Min1-C, two amino groups point outward (N28, N20), i.e., they are pyramidalized outward. This facilitates the H-bonding interaction between two amino groups (NH···NH), in which one acts as H-donor (N9) and another as an H-acceptor (N28). As for Min2, the two moieties interact side by side via a double H-bond of the type NH···N=C. The two molecules are not exactly on the same plane, featuring a dihedral angle of about 33.7°. A second set of minima was obtained by reoptimizing the four structures in the aqueous environment employing the COSMO continuum solvation model^54,55^ with a dielectric constant of 78.36. Overall, the aqueous environment impacts the structures of Min1 and Min2 very slightly, with only some non-negligible changes in the case of Min1-B (see details in the Supporting Information).

### N 1s Binding Energy Simulations

Having identified the main interacting configurations, we calculated the N 1s BEs with DFT (see the Theoretical Calculations Section) for both the MP2/cc-pVDZ-optimized gas-phase and solvated structures. We also calculated BEs for the monomer. The simulated N 1s PE spectra are displayed in the bottom panel of Figure 4. Table 2 collects BEs corresponding to the N 1s components reported in the PE spectra, along with chemical shifts, as shown in the bottom panel of Figure 4. Every component derives by summing up the Gaussian functions associated with the BEs of N atoms which are fully or nearly equivalent. BEs of the individual atoms are reported in the Supporting Information (Table S1).

**Table 2:** N 1s BEs, *cs* and ΔBE_cosmo-gp_ Values Calculated with DFT-B3LYP for Gas-Phase and Solvated MP2/cc-pVDZ-Optimized Structures of Melamine Monomer/Dimers^a^

			**Gas phase**		**COSMO**		
**Structure**	**Molecule**	**N 1s level**	**BE**^b^**(eV)**	***cs***^c^**(eV)**	**BE**^b^**(eV)**	***cs***^c^**(eV)**	d**ΔBE**_**cosmo-gp**_**(eV)**
Monomer	M1	N_t_	403.82	-	401.80	-	–2.02
		N_a_	405.38	1.56	402.82	1.02	–2.56
Min1-A	M1, M2	N_t_	403.74	-	401.87	-	–1.87
		N_a_	405.31	1.57	402.93	1.06	–2.38
Min1-B	M1, M2	N_t_	403.76	-	401.86	-	–1.90
		N_a_-in	405.23	1.53	402.90	1.04	–2.33
		N_a_-out	405.41		402.90		–2.51
Min1-C	M1 H-donor	N_t_	403.51	-	401.86	-	–1.65
	M2 H-acceptor	N_t_	403.83		401.84		–1.99
	M1 H-donor	N_a_	405.00	1.55	402.86	1.01	–2.14
	M2 H-acceptor	N_a_	405.37		402.86		–2.51
Min2	M1, M2	N_t_	403.69	-	401.83	-	–1.86
		N_a_-Hb	404.64	0.95	402.64	0.95	–2.00
		N_a_-*n*Hb	405.34	1.65	402.86		–2.48

a The two melamine moieties are denoted M1 and M2.

b BEs correspond to the N 1s components displayed for each spectrum in Figure 4. Every component derives by summing up the Gaussian functions associated with the BEs of N atoms which are fully or nearly equivalent.

c Chemical shifts, *cs*, correspond to the energy separations between the N_a_ and N_t_ components as shown in Figure 4.

d ΔBE_cosmo-gp_ is the difference between the BE computed with COSMO and the gas phase BE.

Focusing on the gas-phase spectra (left graph of Figure 4), we see that the theoretical curve of the monomer compares well with the experimental one reproducing both the *cs* and the absolute BE values (compare Tables 1 and 2). As for the gas-phase spectra of the dimers, these do not have an experimental counterpart. Nonetheless, the comparison with the monomer allows us to identify the spectral fingerprints of specific intermolecular interactions. The π-stacked dimer, Min1-A, features a chemical shift equivalent to that of the monomer (1.57 eV vs 1.56 eV), revealing that π–π interactions affect in the same way both amino and triazine BEs. In the case of the displaced π-stacked dimer, Min1-B, the outer N_a_ (N_a_-out) have a slightly higher BE (+0.18 eV) compared to the inner ones (N_a_-in). Nonetheless, the average chemical shift, 1.53 eV, is still in line with that of the monomer/Min1-A. More interesting is the case of Min1-C, where π–π interactions are assisted by the formation of an H-bond between two amino groups, one acting as H-donor (N9, M1) and the other as H-acceptor (N28, M2). Similarly to pure π-stacked dimers, the PE spectrum of Min1-C exhibits two main peaks but with lower intensity and larger broadening. In this case, the H-bonding interaction lifts the equivalence between the two molecules, with the H-donor (M1) having lower BE values with respect to those of the H-acceptor (M2): −0.32 eV for N_t_ and −0.37 eV for N_a_. A close inspection of the individual BEs (see Supporting Information) reveals that N9, the H-donor nitrogen, has the lowest N_a_ BE (see Table S1). This leads to the broadening and consequent intensity decrease of both N 1s components. Despite such modifications, we can still identify an average *cs* of 1.55 eV. A more dramatic change is observed in the spectrum of the Min2 dimer. In this case, the two molecules are entirely equivalent, both acting as H-donor and H-acceptor through the double NH···N=C interaction. In these conditions, significant BE variations are observed only for the H-bonding N_a_ atoms (N_a_-Hb), which are shifted by −0.7 eV to lower BEs with respect to the non-H-bonding N_a_ (N_a_-*n*Hb). Consequently, the spectrum is characterized by two N_a_ components with distinct chemical shifts: 0.95 eV for N_a_-Hb and 1.65 eV for N_a_-*n*Hb.

When passing from the gas phase to the solvated structures (right graph of Figure 4), one immediately notices that all spectra feature a similar shape, with a common chemical shift of about 1.0 eV (both monomer and dimers). Indeed, solvation has the effect of suppressing all of the non-equivalences due to the different type of interactions, i.e., compare N_a_-in and N_a_-out (Min1-B), M1 and M2 (Min1-C), and N_a_-Hb and N_a_-*n*Hb (Min2).

To better understand the origin of these effects, we carefully analyzed the BE variation (ΔBE_cosmo-gp_) of the different N atoms when going from the gas phase to the solvated minima, that is, the difference between the COSMO BE and gas-phase BE (see Table 2). In the monomer, the solvation has more impact on the N_a_ BE with respect to the N_t_ one, yielding ΔBEs of −2.56 eV (N_a_) and −2.02 eV (N_t_). This means that the *cs* reduction from 1.56 eV (gas phase) to 1.02 eV (COSMO) is essentially due to the N_a_ 1s level, which decreases by 0.54 eV more than the N_t_ level. Similarly, in the π-stacked dimer, Min1-A, |ΔBE|_N_a__ is larger by 0.51 eV than |ΔBE|_N_t__, yielding a chemical shift of 1.06 eV for the solvated structure. Also for Min1-B, the N_t_ BE shifts by a smaller amount (−1.90 eV) with respect to N_a_-in (−2.33 eV) and N_a_-out (−2.51 eV) BEs. Interestingly, N_a_-out (BE_gp_ = 405.41 eV) experiences a slightly greater ΔBE compared to N_a_-in (BE_gp_ = 405.23), allowing to restore the equivalence among the N_a_ atoms (BE_cosmo_ = 402.90 eV) and an average chemical shift of 1.04 eV. In the case of Min1-S3, within each moiety (H-donor or H-acceptor), the |ΔBE|_N_a__ is always larger than |ΔBE|_N_t__. At the same time, after solvation, the two molecules become perfectly equivalent, featuring almost identical BE values for each type of N. Indeed, the BEs of the H-donor (M1) shift less compared to the corresponding BEs of the H-acceptor (M2), i.e., −1.65 eV and −1.99 for N_t_ of M1 and M2, respectively (see Table 2 for N_a_). In other words, those atoms which already feature significant negative BE variations (in this case, M1) due to the molecule–molecule interactions are less affected by solvation, showing smaller ΔBEs. As a consequence, the equivalence among the same type of N atoms is restored, giving an average *cs* of 1.01 eV. As for Min2, the ΔBEs of both N_a_-*n*Hb and N_a_-Hb are always greater than |ΔBE|_N_t__. However, the BEs of the H-bonding N_a_ (N_a_-Hb) shift less (−2.00 eV) compared to those of the non-interacting ones (−2.48 eV), restoring the equivalence between them and yielding an average *cs* of 0.95 eV.

It is now evident that the spectral features of the molecule–molecule interactions are practically lost in solution, the latter approximated by the COSMO continuum model. Specifically, N 1s BEs are not sensitive to the occurrence of H-bonded dimers or π-stacked configurations, all of them featuring a chemical shift of about 1.0 eV. As a matter of fact, such a value compares well with the experimental *cs* of the N_a1_ component, allowing us to associate it with any kind of solvated amino N (H-bonded or not). Moreover, the experimental ΔBE between the liquid jet N_a1_ and the gas-phase N_a_ components is found to be ∼0.6 eV greater than the BE variation undergone by N_t_, in good agreement with the theoretical trends.

By describing the solvation effects with the COSMO implicit solvation model, we were able to explain the origin of the reduction of the chemical shift in solution, which is mainly due to the lowering of the amino-N BE. However, in the computed spectra, it could not distinguish the two experimental components, N_a1_ and N_a2_. The latter component, presumably originating from additional amino species, can be explained by considering the nature of the μ-LJ technique, which essentially remains a surface-sensitive technique. This means that we are likely probing amino groups at different depths from the liquid/vacuum interface and therefore featuring different degrees of solvation. Since COSMO is a continuum model, only fully solvated melamine molecules were considered, while the effect of a solvation gradient on the N 1s BEs of melamine could not be taken into account. To investigate such an effect, we decided to perform an exploratory classical molecular dynamics simulation comprising one single melamine molecule and 68 explicit water molecules (see the Supporting Information for details). Three random configurations were extracted from the simulation, and BEs were calculated for each N atom by considering explicit waters up to (i) the first solvation shell (13 waters) and (ii) the second one (27 water). The results of this analysis are reported in the Supporting Information. Although the computation of the converged spectra is beyond the scope of the present study, this preliminary calculation confirms that the N_a_ BE is very sensitive to the number of water molecules surrounding the amino group, as also testified in ref (28). By only considering the first solvation shell, the *cs* of the N_a_ component can range from 0.8 eV for amino-N that are well hydrated or strongly H-bonded to water molecules, to 1.4 eV for less hydrated N_a_. Interestingly, the addition of the second shell is not always enough to further reduce the BEs of the scarcely hydrated amino groups, and the *cs* can also jump to 1.7 eV.

## Conclusions

In summary, we have shown how core-level BEs of self-associated melamine molecules in aqueous solution can be satisfactorily calculated and analyzed using an integrated theoretical approach based on classical metadynamics simulations and DFT-based quantum calculations. Through enhanced metadynamics sampling, we have identified four dimeric configurations: two of them exclusively based on π–π interactions (Min1-A, Min1-B), a third one featuring π–π interactions plus an H-bond of the type NH···NH (Min1-C), and a fourth structure (Min2) based on a double H-bonding interaction between the amino-N and the triazine-N, i.e., NH···N=C.

The N 1s PE spectra of the solvated dimers, computed using the COSMO implicit solvation model, were compared with those of the corresponding gas-phase structures. This allowed us to analyze the effect of solvation on each type of N-functional group. Pure π-stacked dimers show PE spectra almost identical to that of the monomer, where the chemical shift between the amino-N (N_a_) and the triazine-N (N_t_) levels notably decreases from ∼1.5 eV in the gas phase to ∼1.0 eV in solution. A more detailed analysis reveals that upon solvation, both levels shift to lower BEs, but the N_a_ level shifts by ∼0.5 eV more with respect to the N_t_ level.

In the case of Min1-C and Min2, the gas-phase spectra bear fingerprints of the corresponding H-bonding interaction. Specifically, the H-bond (NH···NH) existing in Min1-C breaks the equivalence between the H-donor (M1) and H-acceptor (M2) molecules, giving rise to two main components (N_a_, N_t_) separated by 1.55 eV, but having lower intensity and larger broadening compared to those of pure π-stacked dimers. On the other hand, the double H-bond (NH···N=C) of Min2 yields a gas-phase PE spectrum characterized by two amino-N components having a chemical shift of 0.95 eV for the H-bonded N_a_ and 1.5 eV for the non-interacting ones. The inequivalences due to the H-bonds are removed in solution, and the resulting PE spectra are similar to those of the π-stacked dimers with chemical shifts of ca. 1.0 eV. These results compare well with the experimental N 1s PE spectrum obtained for aqueous melamine by means of the XPS μ-LJ technique. Deconvolution of the spectrum, indeed, evidences two main components (N_t_, N_a1_) with a chemical shift of about 0.9 eV, in good agreement with the *cs* of each of the computed dimers. These findings confirm that the COSMO model is a fast and reliable method to predict chemical shifts of fully solvated molecules. Unfortunately, in solution, the signature of intermolecular interactions (π–π or H-bonds) in the core-level BEs and corresponding *cs* is lost. Finally, the surface sensitivity of the XPS μ-LJ allows to evidence the presence of amino species which may not be fully solvated. The latter are indeed contributing to an additional component (N_a2_) in the core PE spectra with a gas-phase-like chemical shift of ca. 1.6 eV.

## Supporting Information Available

Chemical structures of CNH-polymers and monomers; geometrical details of the gas-phase and solvated dimers; a full list of the BEs of the individual N atoms within each dimer; chemical shifts analysis for one melamine molecule considering the first and the second solvation shell of explicit waters, and Cartesian coordinates of the gas-phase and solvated dimeric structure (PDF)
