# Supplementary material for: Inequivalent Solvation Effects on the N 1s Levels of Self-Associated Melamine Molecules in Aqueous Solution
Source: J Phys Chem B. 2023 Mar 27;127(13):3016–25. doi: 10.1021/acs.jpcb.3c00327 (PMC10084451; doi:10.1021/acs.jpcb.3c00327)
Supplement: Supplementary file 1 — jp3c00327_si_001.pdf [file jp3c00327_si_001.pdf]

Supporting Information
Inequivalent Solvation Effects on the N 1s Levels of Self-Associated Melamine Molecules in Aqueous Solution

Aurora Ponzi,[†] Marta Rosa,[‡] Gregor Kladnik,[¶] Isaak Unger,[§] Alessandra Ciavardini,[‖] Lorys Di Nardi,[⊥] Elisa Viola,[⊥] Christophe Nicolas,[#] Nađa Došlić,[†] Andrea Goldoni,[@] and Valeria Lanzilotto[*,⊥,@]

†*Division of Physical Chemistry, Ruđer Bošković Institute, 10000 Zagreb, Croatia*

‡*Department of Chemical Science, University of Padova, 35122 Padova, Italy*

¶*Department of Physics, University of Ljubljana, 1000 Ljubljana, Slovenia*

§*Department of Physics and Astronomy, Uppsala University, 751 20 Uppsala, Sweden*

‖*CERIC-ERIC, 34149 Trieste, Italy*

⊥*Department of Chemistry, Sapienza University of Rome, 00185 Roma, Italy*

#*Synchrotron SOLEIL, 91192 Paris, France*

@*Elettra Synchrotron, Micro & Nano Carbon Laboratory, 34149 Trieste, Italy*

E-mail: valeria.lanzilotto@uniroma1.it,valeria.lanzilotto@units.it

1. CNH-based polymers and monomers

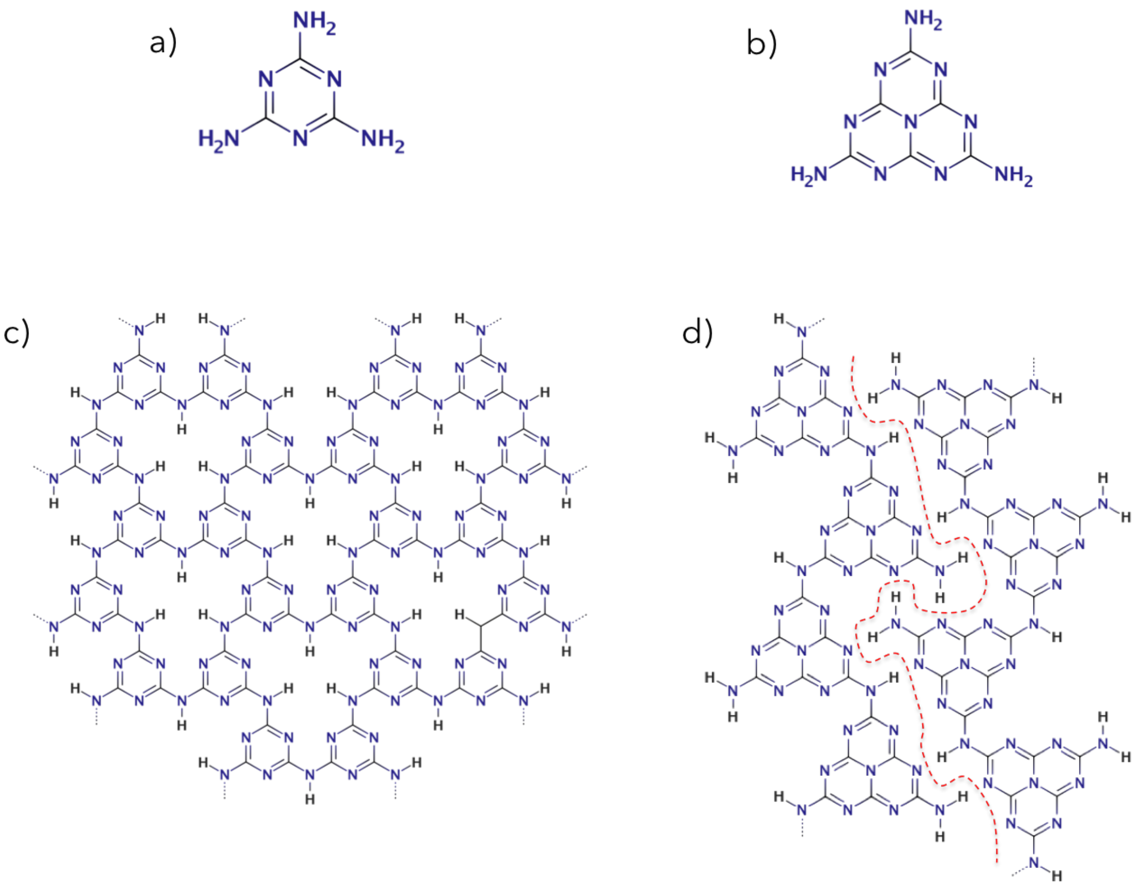

Figure S1: Chemical structure of a) melamine (triamino-s-triazine), b) melem (triamino-s-heptazine), c) poly-triazine-imide (PTI), a graphitic-like melamine-based polymer and d) an H-bonded 2D assembly of melon chains, a liner polymer based on the melem unit.

2. Dimers

2.1 Geometrical details

In Min1-A one ring is on top of the other, while in Min1-B and Min1-C the two rings are slid by 1.4 and 1.3 Å, respectively (see Figure S2 for the definition of the coordinates). Thus, the amino groups in Min1-A are almost equivalent. The NH_2 groups are piramidalized on average by +/-29.7°and all hydrogen atoms point inward. The NH_2 groups in Min1-B and Min1-C are inequivalent and have piramidalization angles in the range +/-27 to +/-34.8°.

As for Min2, the two molecules are not on the same plane as they are characterized by a rise of 0.5 Åand their rings are tilted by 37.5°. This results in very inequivalent NH_2 groups – those engaged in a hydrogen bonding interaction are piramidalized on average by 17.4°, while free NH_2 are piramidalized by 27.9°.

The aqueous environment impacts very slightly the structure of Min1-A. The separation of 3.12 Åbetween the two rings is reduced from 41.9 to 40.3°and the NH_2 groups are less piramidalized (+/- 28.7°). The same applies for Min1-C whose structure is practically unchanged in solution. The COSMO environment affects more Min1-B, where the slide between the two rings is reduced by 0.1 Å. This in turn affects the separation between the two rings, which is 3.1 Åin solution. As in Min1-A and Min1-C, the piramidalization angle of the NH_2 groups is reduced in solution by approximately 1.0°. Finally, the COSMO environment affects only slightly the hydrogen bonded Min2. The two hydrogen bond distances are virtually unchanged (3.0 Å) but the rise coordinate increased to 1.0 Å. This resulted in a more pronounced piramidalization (21.3°) of the NH_2 groups engaged in the hydrogen bonding interaction and slight decrease in the piramidalization of the free NH2 groups (26.8°).

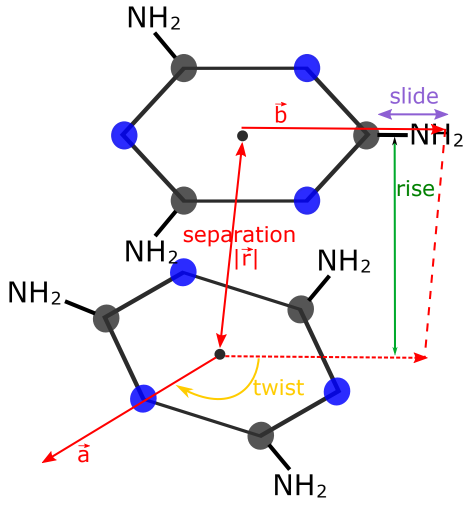

Figure S2: Definition of the slide, rise, separation and twist coordinates. Rise and slide coordinates are defined as the vertical and horizontal separation between the midpoints of the base rings (indicated as black circles), respectively. The length of the vector $\vec{r}$ connecting the geometrical centers of melamine rings defines the melamine-melamine separation. The twist angle is the angle between vector $\vec{a}$ belonging to the lower melamine and a projection of the vector $\vec{b}$ on the plane defined by the atoms of the lower melamine ring.

2.2 Individual Binding Energies

Table S1: Theoretical N 1s BEs of the gas-phase and solvated dimer structures.

		**Min1-A**		**Min1-B**		**Min1-C**		**Min2**	
		BE_{gp}	BE_{cosmo}	BE_{gp}	BE_{cosmo}	BE_{gp}	BE_{cosmo}	BE	BE_{cosmo}
M1	N_a-5	405.31	402.93	405.22	402.90	405.20	402.85	404.62	402.65
	N_a-9	405.31	402.93	405.23	402.90	404.77	402.81	405.30	402.82
	N_a-13	405.30	402.93	405.41	402.90	405.03	402.91	405.37	402.90
M2	N_a-20	405.31	402.93	405.24	402.90	405.45	402.82	405.38	402.89
	N_a-24	405.31	402.93	405.41	402.90	405.24	402.92	405.32	402.82
	N_a-28	405.31	402.93	405.23	402.91	405.43	402.85	404.67	402.64
M1	N_t-2	403.74	401.87	403.78	401.86	403.58	401.88	403.72	401.91
	N_t-4	403.75	401.87	403.71	401.86	403.49	401.84	403.64	401.79
	N_t-12	403.74	401.87	403.79	401.86	403.45	401.85	403.69	401.81
M2	N_t-17	403.75	401.87	403.72	401.86	403.87	401.84	403.73	401.91
	N_t-19	403.74	401.87	403.79	401.86	403.83	401.83	403.71	401.81
	N_t-27	403.74	401.86	403.79	401.86	403.81	401.84	403.66	401.79

3. Explicit waters models

3.1 Chemical shifts analysis

In order to preliminary investigate the effect of a solvation gradient on the N 1s BEs of the melamine molecule, we decided to perform a DFT-based molecular dynamics (DFT-MD) simulation comprising one single melamine molecule and 68 explicit water molecules. Thus, three random configurations were extracted and BEs were calculated for each N atom by considering explicit waters up to (i) the first solvation shell (13 waters) and (ii) the second one (27 water).

The three random DFT-MD based configurations are shown in Figure S3 along with the first solvation shell. Geometrical parameters of each water-melamine H-bond (or pseudo H-bond) are given in Table S2.

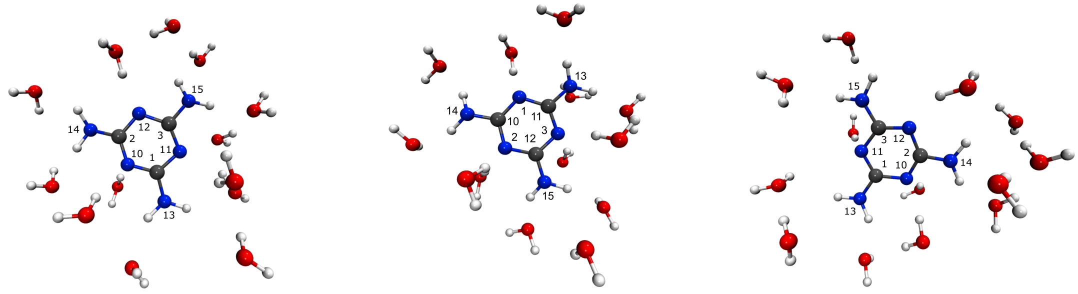

Geom 1 Geom 2 Geom 3

Figure S3: The three random 13-waters-1-melamine configurations extracted from DFT-MD simulation of one melamine solvated by 68 waters.

Table S2: H-bond/pseudo H-bond parameters (distance and angle) between the closest water molecules and the N-functionalities of the melamine molecule in the three "explicit-waters" configurations.

Geom	H-bond	Distance (Å)	Angle (°)
Geom1-NC	N10$\cdots$H-O36/N10$\cdots$H-O33	2.05/2.09	166/153
	N11$\cdots$H-O27/N11$\cdots$H-O51	2.45/2.47	123/138
	N12$\cdots$H-O39/ -	1.86/ -	169/ -
Geom1-NH_2	N13-H$\cdots$O45/N13-H$\cdots$O27	2.22/2.33	151/145
	N14-H$\cdots$O21/N14-H$\cdots$O18	2.16/2.33	168/129
	N15-H$\cdots$O30/N15-H$\cdots$O42	1.95/2.51	162/156
Geom2-NC	N10$\cdots$H-O33/ -	1.65/ -	166/153
	N11$\cdots$H-O54/ -	1.92/ -	123/138
	N12$\cdots$H-O36/N12$\cdots$H-O42	1.79/1.84	169/ -
Geom2-NH_2	N13-H$\cdots$O27/N13-H$\cdots$O39	1.87/1.89	162/165
	N14-H$\cdots$O21/N14-H$\cdots$O18	1.87/2.03	155/158
	N15-H$\cdots$O48/N15-H$\cdots$O24	1.83/2.12	145/159
Geom3-NC	N10$\cdots$H-O45/N10$\cdots$H-O42	1.80/2.00	157/171
	N11$\cdots$H-O54/ -	2.24/ -	178/138
	N12$\cdots$H-O39/ -	2.11/ -	148/ -
Geom3-NH_2	N13-H$\cdots$O48/N13-H$\cdots$O36	1.97/2.59	151/171
	N14-H$\cdots$O21/N14-H$\cdots$O39	2.12/2.52	150/132
	N15-H$\cdots$O33/N15-H$\cdots$O30	2.29/2.60	153/112

N 1s PE spectra for the three different geometries are reported in Figure S4 by considering the first (bottom curves) and the second solvation shell (top curves). Table S3 collects BEs and *cs* corresponding to the N 1s components reported in the PE spectra. Every component derives by summing up the Gaussian functions associated with the BEs of N atoms which

are fully or nearly equivalent. BEs of the individual atoms are reported in the following subsection.

Table S3: BEs and *cs* corresponding to the N 1s components displayed for each PE spectrum in Figure S4. Every component derives by summing up the Gaussian functions associated with the BEs of N atoms which are fully or nearly equivalent.

Geom	N 1s level	1st shell BE	1st shell *cs*	2nd shell BE	2nd shell *cs*
Geom1	N_t	403.61		403.69	
	N_{a1}	404.32	0.71	404.41	0.72
	N_{a2}	404.94	1.33	404.92	1.23
Geom2	N_t	403.20		403.83	
	N_{a1}	404.03	0.83	404.27	0.44
	N_{a2}			405.11	1.28
Geom3	N_t	403.67		403.36	
	N_{a1}	404.47	0.80	404.24	0.88
	N_{a2}	405.08	1.41	405.06	1.70

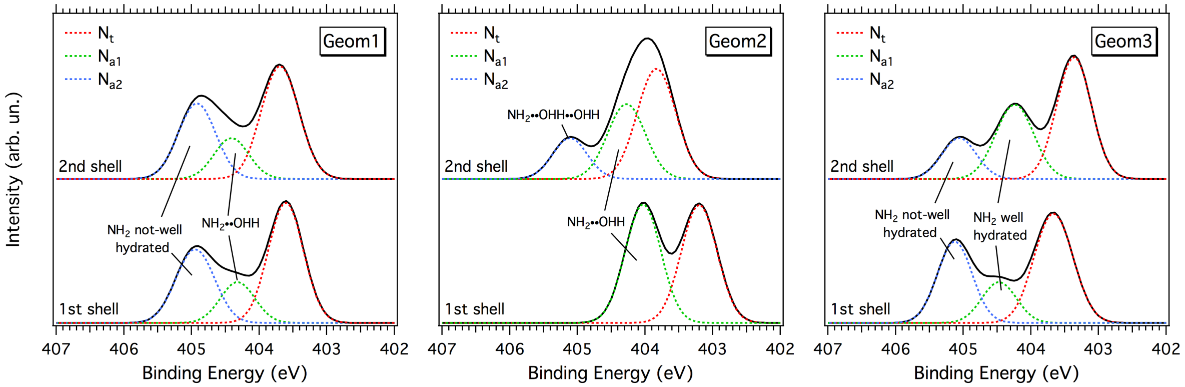

Figure S4: Simulated N 1s PE spectra of the three random geometries extracted from the DFT-MD comprising one melamine molecule and 68 explicit waters. The spectra were computed by considering up to the first solvation shell (13 water moelcules) and the second one (27 water molecules).

3.2 Individual BEs values - first and second solvation shell

Table S4: N 1s BE values calculated for three random configurations extracted from DFT-MD simulations by considering explicit waters up to the first solvation shell (13 water molecules). Molpro details: ΔSCF/B3LYP/def2-TZVPP (for C and H), def2-QZVPP (for N). Absolute and relative energies are reported in eV.

	**Geom1**	**Geom2**	**Geom3**
N_a-13	404.83	403.96	405.10
N_a-14	405.06	404.12	404.47
N_a-15	404.32	404.01	405.15
N_t-10	403.66	403.28	403.50
N_t-11	403.62	403.10	403.69
N_t-12	403.53	403.21	403.80

Table S5: N 1s BE values calculated for three random configurations extracted from DFT-MD simulations by considering explicit waters up to the second solvation shell (27 water molecules). Molpro details: ΔSCF/B3LYP/def2-TZVPP (for C and H), def2-QZVPP (for N). Absolute and relative energies are reported in eV.

	**Geom1**	**Geom2**	**Geom3**
N_a-13	404.82	405.11	405.06
N_a-14	405.02	404.17	404.35
N_a-15	404.41	404.38	404.12
N_t-10	403.72	403.84	403.38
N_t-11	403.80	403.97	403.33
N_t-12	403.55	403.68	403.38

3.3 Computational details

Ground state density functional theory-based molecular dynamics of melamine in aqueous environment is performed with the BLYP functional[1,2] along with the Grimme's D3 correction for the dispersion interactions.[3] We used the DZVP basis set optimized for calculations in condensed phase[4] for valence electrons and GTH type pseudopotentials[5] for core electrons. Plane waves are also included in calculation via the mixed Gaussian and plane waves (GPW) method[6] with the cutoff set to 360 Ry. Melamine is solvated with 68 water molecules under

periodic boundary conditions which yielded a orthorhombic unit cell with lengths of 12.17, 15.08 and 11.550 Å in x, y and z directions, respectively. System was equilibrated in the canonical ensemble for 2 ps to the temperature of 300 K using the CSVR thermostat[7] and the production run lasted for 15 ps under same conditions. Simulation box size and initial geometry and velocities are determined from the force field NPT molecular dynamics simulation. AmberTools20 program package[8] was used to generate topology with GAFF[9] and TIP3P[10] parameters set for melamine and water molecules, respectively. Atomic charges were obtained using the AM1-BCC model. In all molecular dynamics simulations we used 0.5 fs timestep for integrating classical equations of motion. All molecular dynamics calculations are performed using the CP2K program package.[11]

4. Geometries - dimers coordinates

Table S6: Cartesian coordinates of MP2/cc-pVDZ optimized gas-phase and solvated structures of the melamine monomer.

Gas-phase monomer				Solvated monomer			
C	-0.3326129	1.2574486	-0.0220838	C	-0.3315020	1.2519755	-0.0237051
N	0.9866147	0.9805497	-0.0000537	N	0.9862081	0.9802465	-0.0024069
C	1.2556535	-0.3403896	0.0133641	C	1.2499767	-0.3388300	0.0155561
N	0.3563331	-1.3446164	-0.0001194	N	0.3561534	-1.3440812	-0.0024826
C	-0.9221484	-0.9173144	-0.0221773	C	-0.9184392	-0.9131229	-0.0237846
N	-1.3420963	0.3637984	-0.0296555	N	-1.3425138	0.3639412	-0.0222763
N	2.5718565	-0.6971539	0.1048381	N	2.5710769	-0.6969328	0.1100848
N	-1.8889134	-1.8804692	-0.0953878	N	-1.8876497	-1.8801943	-0.0961126
N	-0.6805875	2.5769872	-0.0951619	N	-0.6796550	2.5761950	-0.0959359
H	3.2345154	0.0114742	-0.1958771	H	3.2216392	0.0179112	-0.1967215
H	2.7860232	-1.6432888	-0.1962133	H	2.7715470	-1.6424829	-0.1967694
H	-2.8164268	-1.5871365	0.1967391	H	-2.8102741	-1.5773097	0.1951771
H	-1.6072400	-2.8025814	0.2241245	H	-1.5940473	-2.7964406	0.2236860
H	0.0283431	3.2306360	0.2239948	H	0.0365151	3.2186623	0.2239196
H	-1.6291793	2.7922801	0.1972513	H	-1.6289001	2.7806868	0.1953531

Table S7: Cartesian coordinates of MP2/cc-pVDZ optimized gas-phase and solvated structures of the melamine Min1-A dimer.

	**Gas-phase Min1-A dimer**				**Solvated Min1-A dimer**		
C	34.2472319	5.0922982	95.2393066	C	34.2711592	5.0836396	95.2354111
N	33.0026875	5.5491964	95.4729515	N	33.0304220	5.5535419	95.4743030
C	32.0466876	4.6841485	95.0914453	C	32.0578862	4.6975867	95.1076435
N	32.2248363	3.4745402	94.5280700	N	32.2220025	3.4877004	94.5364195
N	30.7479480	5.0929638	95.2642867	N	30.7679898	5.1198663	95.2900989
H	30.6421944	5.8367293	95.9456414	H	30.6609587	5.8557618	95.9824757
H	30.0829697	4.3308406	95.3442728	H	30.0847153	4.3709746	95.3619382
C	33.5214552	3.1608691	94.3599824	C	33.5144763	3.1543510	94.3585644
N	33.7863137	1.9555556	93.7591982	N	33.7600894	1.9517087	93.7522407
H	34.6983636	1.5734595	93.9864686	H	34.6763619	1.5581415	93.9479414
H	33.0147553	1.2988910	93.8048315	H	32.9932596	1.2875003	93.8071835
N	34.5877262	3.9148271	94.6867650	N	34.5906362	3.9026881	94.6732657
N	35.2798751	5.9340425	95.5702645	N	35.3128589	5.9135015	95.5538571
H	35.0508059	6.5953084	96.3050341	H	35.1038380	6.5950391	96.2781779
H	36.1710563	5.4611019	95.6747646	H	36.2071959	5.4435678	95.6618631
C	34.6427035	3.0118586	97.7107182	C	34.6375137	3.0307729	97.7090346
N	34.0586789	4.1073616	98.2271314	N	34.0331459	4.1145511	98.2315171
C	32.7142521	4.0381986	98.2189003	C	32.6876455	4.0326871	98.2162985
N	31.9577856	3.0233059	97.7655757	N	31.9484349	3.0027269	97.7626419
N	32.0435389	5.1050025	98.7630331	N	32.0033766	5.0852844	98.7624932
H	31.0740390	5.1670880	98.4716271	H	31.0320666	5.1529686	98.4724014
H	32.5630421	5.9760665	98.7337290	H	32.5088987	5.9668367	98.7594953
C	32.6872615	2.0014152	97.2799920	C	32.6906984	1.9905696	97.2708153
N	31.9869821	0.9106210	96.8281878	N	32.0084124	0.8917276	96.8212371
H	31.0444886	1.1263953	96.5203342	H	31.0537610	1.0779729	96.5267176
H	32.5214522	0.3146453	96.2053625	H	32.5372485	0.2980739	96.1885751
N	34.0279156	1.9195072	97.2198273	N	34.0347107	1.9289206	97.2193756
N	36.0149907	2.9903963	97.7153131	N	36.0067916	3.0264716	97.7199055
H	36.4204371	2.3847272	97.0094777	H	36.4329621	2.4177608	97.0266364
H	36.4375795	3.9086413	97.7976224	H	36.4305457	3.9471090	97.7916087

Table S8: Cartesian coordinates of MP2/cc-pVDZ optimized gas-phase and solvated structures of the melamine Min1-B dimer.

**Gas-phase Min1-B dimer**				**Solvated Min1-B dimer**			
C	33.6810211	4.7933962	96.9436010	C	33.7536245	4.4339365	97.2000316
N	32.6183420	4.0435221	97.2902457	N	32.6833756	3.6480301	97.4285861
C	32.9127546	2.7351157	97.4044696	C	32.9545070	2.3330239	97.3170935
N	34.1108271	2.1551259	97.2146485	N	34.1415135	1.7790454	97.0022567
N	31.8688096	1.8925205	97.7045558	N	31.9028819	1.4731709	97.4925080
H	31.0735820	2.3723833	98.1129388	H	31.1418531	1.8510297	98.0502465
H	32.1622992	1.0583799	98.2053202	H	32.1747249	0.5279862	97.7484452
C	35.0729906	3.0332332	96.8815730	C	35.1160553	2.6875726	96.7996173
N	36.3166216	2.5063262	96.6266398	N	36.3412344	2.2000728	96.4306527
H	37.0589781	3.1971668	96.6568362	H	37.1156602	2.8345057	96.6053071
H	36.5050909	1.6558343	97.1498113	H	36.5144399	1.2405132	96.7173199
N	34.9388055	4.3639628	96.7299732	N	34.9964615	4.0273542	96.8743796
N	33.4505787	6.1302742	96.7538720	N	33.5449970	5.7851653	97.2550166
H	32.6165971	6.4883669	97.2051415	H	32.7403264	6.0682048	97.8071731
H	34.2753376	6.7174129	96.8029112	H	34.3824993	6.3369679	97.4175919
C	35.7231512	3.3223890	99.8261963	C	35.7174429	3.6735188	99.8972982
N	34.5265414	3.9033200	100.0222259	N	34.5589224	4.2888960	100.2051902
C	33.5630369	3.0246153	100.3495265	C	33.5414935	3.4319061	100.4181613
N	33.6943288	1.6922346	100.4888176	N	33.5949958	2.0868664	100.3588914
N	32.3215295	3.5524108	100.6118413	N	32.3417584	3.9826533	100.7822698
H	31.5760851	2.8652853	100.5761995	H	31.5366394	3.3887197	100.6041526
H	32.1353087	4.4109288	100.1014153	H	32.2186408	4.9483808	100.4907977
C	34.9503941	1.2615709	100.2676051	C	34.8163704	1.6160662	100.0394883
N	35.1770172	-0.0779965	100.4423115	N	34.9595775	0.2558969	100.0021200
H	34.3499589	-0.6616033	100.3890554	H	35.7469882	-0.0718726	99.4499635
N	36.0145996	2.0121809	99.9271784	N	35.9239524	2.3458131	99.8019232
N	36.7683896	4.1659506	99.5343415	N	36.8095021	4.4786390	99.7125331
H	37.5628884	3.6910419	99.1190923	H	37.5566196	4.0540376	99.1701466
H	36.4762546	5.0088356	99.0478420	H	36.5868021	5.4318298	99.4396104

Table S9: Cartesian coordinates of MP2/cc-pVDZ optimized gas-phase and solvated structures of the melamine Min1-C dimer.

**Gas-phase Min1-C dimer**				**Solvated Min1-C dimer**			
C	17.9631521	4.3181995	95.8197197	C	17.9512801	4.3037834	95.8196082
N	18.4476812	5.3878178	95.1604858	N	18.4379465	5.3634669	95.1469720
C	17.8529394	5.5779729	93.9656955	C	17.8307629	5.5673199	93.9597120
N	16.8518481	4.8573379	93.4356883	N	16.8250536	4.8395298	93.4378801
N	18.3463695	6.6001846	93.1934269	N	18.3175699	6.5869198	93.1887129
H	18.8579737	7.3093982	93.7062971	H	18.8323691	7.3021330	93.6943515
H	17.7025010	6.9382938	92.4866652	H	17.6742388	6.9378497	92.4850851
C	16.4519943	3.8457327	94.2282960	C	16.4284469	3.8283818	94.2323315
N	15.4297646	3.0579131	93.7560866	N	15.4151574	3.0273958	93.7566596
H	14.9135434	2.6169868	94.5141299	H	14.8873452	2.5944843	94.5130010
H	14.8297096	3.5709111	93.1157061	H	14.8055526	3.5293907	93.1138128
N	16.9728877	3.5028182	95.4212829	N	16.9580962	3.4853679	95.4221075
N	18.5655042	4.0029701	97.0157774	N	18.5616863	3.9900491	97.0091267
H	17.9396040	3.5342599	97.6652900	H	17.9431121	3.5243630	97.6697173
H	19.1014226	4.7678118	97.4120131	H	19.0898178	4.7579500	97.4155431
C	14.1909299	5.3308891	95.6899477	C	14.2132046	5.3537057	95.6853620
N	13.8449105	4.2222070	96.3694263	N	13.8722628	4.2349757	96.3567705
C	14.5622018	4.0517388	97.4960528	C	14.5820810	4.0517755	97.4874440
N	15.5237939	4.8635882	97.9721143	N	15.5366500	4.8678515	97.9781599
N	14.3146086	2.9117688	98.2131625	N	14.3407189	2.9029544	98.1873150
H	13.4157635	2.4798291	98.0302130	H	13.4386673	2.4709126	98.0091273
H	14.6040349	2.9491119	99.1841781	H	14.6153793	2.9311775	99.1651293
C	15.7628949	5.9211499	97.1760213	C	15.7744014	5.9385979	97.1972502
N	16.7066072	6.8205072	97.6125106	N	16.6965221	6.8444689	97.6591251
H	17.3485338	6.4162432	98.2867852	H	17.3594609	6.4572284	98.3249737
H	17.1678683	7.3064582	96.8481228	H	17.1287280	7.4009384	96.9259924
N	15.1367764	6.2243730	96.0231576	N	15.1551946	6.2494308	96.0424287
N	13.5289163	5.5496222	94.5025955	N	13.5682246	5.5797523	94.5024273
H	13.5815620	6.5109348	94.1800024	H	13.6136588	6.5347754	94.1601943
H	12.6035914	5.1329301	94.4684876	H	12.6662990	5.1230301	94.4030172

Table S10: Cartesian coordinates of MP2/cc-pVDZ optimized gas-phase and solvated structures of the melamine Min2 dimer.

**Gas-phase Min2 dimer**				**Solvated Min2 dimer**			
C	19.7543638	26.7230211	95.2992327	C	19.7503008	26.7174659	95.2299686
N	20.2038337	25.4668422	95.4716786	N	20.2004175	25.4719249	95.4830490
C	19.2162013	24.5438902	95.5734977	C	19.2169165	24.5521846	95.6311033
N	17.8939122	24.7908893	95.4797451	N	17.8925080	24.7887472	95.5288067
N	19.6008255	23.2574547	95.7514370	N	19.6075519	23.2732464	95.8603119
H	20.5394926	23.0827019	96.1319345	H	20.5518300	23.1336861	96.2419198
H	18.8544397	22.6118188	95.9801299	H	18.8766405	22.6453663	96.1808193
C	17.6057045	26.0841975	95.2590567	C	17.5917216	26.0717866	95.2485733
N	16.2836346	26.3921605	95.0690157	N	16.2723021	26.3643494	95.0559208
H	16.0483325	27.3645869	95.2330621	H	16.0220555	27.3435014	95.1562084
H	15.6342495	25.7048615	95.4348778	H	15.6148352	25.7029043	95.4578910
N	18.4741438	27.1106066	95.1715522	N	18.4635157	27.0900754	95.0934784
N	20.6950364	27.7209258	95.3011509	N	20.6871554	27.7103259	95.1608447
H	20.3954466	28.5862492	94.8664569	H	20.3888914	28.5515227	94.6755301
H	21.6469878	27.4286712	95.1135001	H	21.6346660	27.4112366	94.9514554
C	23.3373780	23.3422041	95.9078532	C	23.3802124	23.4096493	96.0110388
N	22.3948513	22.8397344	96.7429985	N	22.4274866	22.8804580	96.8163902
C	22.7942957	21.7430160	97.4100758	C	22.7869295	21.7185303	97.3977617
N	23.9878564	21.1357446	97.3259983	N	23.9641210	21.0824431	97.2553245
N	21.8568488	21.1523421	98.2287937	N	21.8355455	21.0983455	98.1620370
H	21.1619481	21.7999987	98.5873771	H	21.1272496	21.7155629	98.5495750
H	22.2665567	20.5426534	98.9297067	H	22.1984405	20.4126319	98.8186459
C	24.8184580	21.7362503	96.4480539	C	24.8178647	21.7188093	96.4240731
N	26.0733498	21.1972301	96.3413331	N	26.0522886	21.1558606	96.2745657
H	26.5726709	21.4481921	95.4955282	H	26.5694784	21.4478676	95.4508149
H	26.1489329	20.2322621	96.6424392	H	26.1126509	20.1675563	96.5003052
N	24.5677053	22.8257636	95.7055130	N	24.5958034	22.8742822	95.7689791
N	23.0159760	24.4690227	95.2361131	N	23.0923642	24.5983802	95.4297438
H	23.6385329	24.7331377	94.4833634	H	23.6887812	24.8728702	94.6556928
H	22.0478813	24.8134078	95.2678620	H	22.1093208	24.8982646	95.4185034

References

(1) Becke, A. D. Density-Functional Exchange-Energy Approximation with Correct Asymptotic-Behavior. *Phys. Rev. A* **1988**, *38*, 3098–3100.

(2) Lee, C. T.; Yang, W. T.; Parr, R. G. Development of the Coll-Salvetti Correlation-Energy Formula into a Functional of the Electron-Density. *Phys. Rev. B* **1988**, *37*, 785–789.

(3) Grimme, S.; Antony, J.; Ehrlich, S.; Krieg, H. A Consistent and Accurate Ab Initio Parametrization of Density Functional Dispersion Correction (DFT-D) for the 94 Elements H-Pu. *J. Chem. Phys.* **2010**, *132*, 154104.

(4) VandeVondele, J.; Hutter, J. Gaussian basis sets for accurate calculations on molecular systems in gas and condensed phases. *J. Chem. Phys.* **2007**, *127*, 114105.

(5) Goedecker, S.; Teter, M.; Hutter, J. Separable Dual-Space Gaussian Pseudopotentials. *Phys. Rev. B* **1996**, *54*, 1703–1710.

(6) Lippert, G.; Hutter, J.; Parrinello, M. A Hybrid Gaussian and Plane Wave Density Functional Scheme. *Mol. Phys.* **1997**, *92*, 477–487.

(7) Bussi, G.; Donadio, D.; Parrinello, M. Canonical Sampling Through Velocity Rescaling. *J. Chem. Phys.* **2007**, *126*, 014101.

(8) Case, D. A. et al. amber 2020. University of California, San Francisco. **2020**,

(9) Wang, J.; Wolf, R. M.; Caldwell, J. W.; Kollman, P. A.; Case, D. A. Development and testing of a general amber force field. *J. Comp. Chem.* **2004**, *25*, 1157–1174.

(10) Jakalian, A.; Jack, D. B.; Bayly, C. I. Fast, efficient generation of high-quality atomic charges. am1-bcc model: Ii. parameterization and validation. *J. Comp. Chem.* **2002**, *23*, 1623–16414.

(11) VandeVondele, J.; Krack, M.; Mohamed, F.; Parrinello, M.; Chassaing, T.; Hutter, J. QUICKSTEP: Fast and Accurate Density Functional Calculations using a Mixed Gaussian and Plane Waves Approach. *J. Comp. Phys. Comm.* **2005**, *167*, 103–128.
